# Association of *TNF-α* promoter polymorphism and Graves’ disease: an updated systematic review and meta-analysis

**DOI:** 10.1042/BSR20180143

**Published:** 2018-03-21

**Authors:** Yaqin Tu, Guorun Fan, Tianshu Zeng, Xiong Cai, Wen Kong

**Affiliations:** 1 Department of Otorhinolaryngology, Union Hospital, Tongji Medical College, Huazhong University of Science and Technology, 1277 Jiefang Ave., Wuhan 430022, China; 2 Department of Endocrinology, Union Hospital, Tongji Medical College, Huazhong University of Science and Technology, 1277 Jiefang Ave., Wuhan 430022, China; 3 Department of Hepatobiliary Surgery, Union Hospital, Tongji Medical College, Huazhong University of Science and Technology, 1277 Jiefang Ave., Wuhan 430022, China

**Keywords:** Graves’ disease, Polymorphism, Tumor Necrosis Factor-α

## Abstract

Graves’ disease (GD) is a common autoimmune disorder with a genetic predisposition. Owing to the biological effect of tumor necrosis factor-α (TNF-α) on the thyroid gland and its gene location, TNF-α should be able to influence an individual’s susceptibility to GD. In the present study, we conduct a meta-analysis of rs1800629 and rs361525 in *TNF-α* gene from all eligible case–control studies to assess the associations amongst reported *TNF-α* gene with GD. A total of ten case–control studies involving 2790 GD patients and 3472 healthy controls were included. The results showed that a significant association was characterized between the rs1800629 polymorphism and GD in the homozygous model (AA compared with GG: odds ratio (OR) = 1.97, 95% confidence interval (CI) = 1.27–3.06, *P*=0.002) and recessive model (AA compared with GA + GG: OR = 1.62, 95% CI = 1.04–2.50, *P*=0.03). GD susceptibility was significantly detected in European population in all genetic models after ethnicity stratification. In sharp contrast, no significant association could be detected in Asian population. Next, we conducted a meta-analysis for another promoter SNP rs361525. However, SNP rs361525 did not show a significant association with GD in any genetic model before and after ethnicity stratification. Together, our data support that only the promoter single-nucleotide polymorphism (SNP) rs1800629 within the *TNF*-α gene is associated with increased risk for developing GD, especially in European population. Future large-scale studies are required to validate the associations between *TNF-α* gene and GD.

## Introduction

Graves’ disease (GD) is an autoimmune thyroid disease with a 0.5% rate of prevalence in general population [1]. It is characterized by the presence of thyroid-stimulating hormone (TSH) receptor antibodies, leading to hyperthyroidism and goiter. The exact etiology of GD has still remained unknown; however, it is believed that genetic polymorphisms and environmental factors are both involved in the pathogenesis of GD. Since GD is an autoimmune disorder, it is affected by genes, cytokines, and enzymes [2]. Genome-wide scans have identified the human leukocyte antigen (HLA) genomic region of the MHC on chromosome 6p21 linked to GD [3,4]. Tumor necrosis factor-α (TNF-α), residing in the short arm of human chromosome 6 (6p21.3), contains genes encoding HLA molecules. Owing to the biological effect of TNF-α on the thyroid gland and its gene location, TNF-α should be able to affect an individual’s susceptibility to GD [5]. Therefore, *TNF-α* gene is a functional candidate for studying GD.

**Figure 1:**
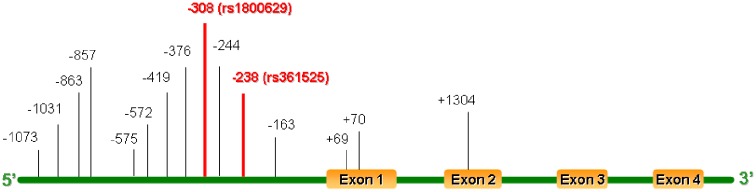
SNPs in the human *TNF-α* gene

Full-length human *TNF-α* gene spans 2.76-kb DNA, with four exons and three introns. Single-nucleotide polymorphisms (SNPs) within *TNF-α* have a potential to cause structural changes within regulatory sites that could affect the function or regulation of TNF-α production. These factors could contribute to the autoimmune process making it an ideal candidate for the development of GD [6]. The *TNF-α* gene has been noted to be very polymorphic as manifested by the enrichment of many exonic, intronic as well as promoter SNPs (Figure 1) [7]. Although the mechanisms underlying TNF-α modulation of the risks for GD are yet to be fully addressed, elucidation of its genetic predisposition for GD, however, may offer some important clues. Indeed, several variations in the promoter region of the *TNF-α* gene have been suggested to be associated with increased risks to the development of GD by several genome-wide association studies (GWAS) [8,9]. Particularly, the most widely investigated SNPs of the *TNF-α* are G-238A (rs361525) and G-308A (rs1800629) in the promoter region, both of them are G to A substitutions. Although similar meta-analyses for the same SNP have already been conducted by Li et al. [10] ~10 years ago, these studies never were comprehensive and the outcomes were found to be conflicting results as well. We, therefore, in the current report, conducted an updated meta-analysis of SNPs rs361525 and rs1800629 in *TNF-α* gene from all eligible case–control studies to assess the associations amongst reported *TNF-α* gene with GD.

## Methods

### Eligible studies

PubMed, Embase, and ISI Web of Science were searched (the last search was conducted on 25 December, 2017) using the following search terms: ‘TNF-α OR Tumor necrosis factor-alpha’, ‘polymorphism OR variant OR mutation’, and ‘Graves’ disease’. References, which were listed in each identified article, were also searched manually to identify additional eligible studies.

### Validity assessment

To be eligible, the following inclusion criteria were established: (i) a human case–control study of a polymorphism associated with GD; (ii) studies that included sufficient genotype data for extraction. Main exclusion criteria for studies were as follows: (i) case reports, letters, reviews, and editorial articles; (ii) literature not containing information regarding diabetes research; (iii) study involving only a case population; and (iv) study not written in English. In the case of multiple studies by the same researchers involving the same or overlapping datasets, we selected the most recent study with the largest number of participants.

### Data extraction and quality assessment

Two curators (Y.T. and G.F.) independently extracted information from included studies. Disagreement was resolved by discussion between the two authors. The following data were extracted: first author’s name, year of publication, ethnicities of the individuals involved, the genotyping method, number of cases and controls for each genotype, and the Hardy–Weinberg equilibrium (HWE) amongst the controls. Ethnicity was categorized as Asian and European. A double-check procedure was performed to ensure accuracy of data entry. To evaluate the study quality, we adopted the Newcastle–Ottawa Scale (NOS) with a nine-star system; this scale assesses the quality of cohort and case–control studies. NOS focusses on three separate sections of stars representing the assessment score. The maximal score of NOS is 9 stars: 4 stars for the selection process, 2 stars for comparability, and 3 stars for exposure/outcome. A score of 7 and above was considered to be high-quality study.

### Statistical analysis

The strength of associations between SNPs rs1800629 and rs361525 within the *TNF-α* gene and the risks for GD was assessed by odds ratios (ORs) with 95% confidence intervals (CIs). We explored the association between rs1800629 and GD in homozygote model (AA compared with GG), heterozygote model (GA compared with GG), dominant model (AA + GA compared with GG), recessive model (AA compared with GA + GG), and additive model (A compared with G), respectively. The same genetic models were applied for SNP rs361525 as well. Chi-squared-based Q-statistic test was employed to assess the between-study heterogeneity, and in any case *P*<0.10 was considered with significant heterogeneity between datasets. Once the effects were assumed to be homogeneous, fixed-effects model was then applied (the Mantel–Haenszel method); otherwise, the random-effects model (DerSimonian and Laird method) was employed appropriately. Sensitivity analysis was performed to assess the influence of each individual study by omitting one study at a time and calculating a pooled estimate for the remainder of the studies. The inverted funnel plots and Egger’s regression test were used to investigate publication bias. Potential publication bias was assessed with funnel plots of the effect sizes compared with the S.E.M.; Begg’s test was used to identify significant asymmetry. If there is evidence of publication bias, funnel plot is noticeably asymmetric. Concerning the significance level of the Begg’s and Egger’s tests was set at 0.05. All statistical tests carried out in the present report were two-tailed. All analyses were conducted using the STATA 11.0 software (STATA Corporation, College Station, TX, U.S.A.).

## Results

### Workflow for the identification of eligible datasets

**Figure 2:**
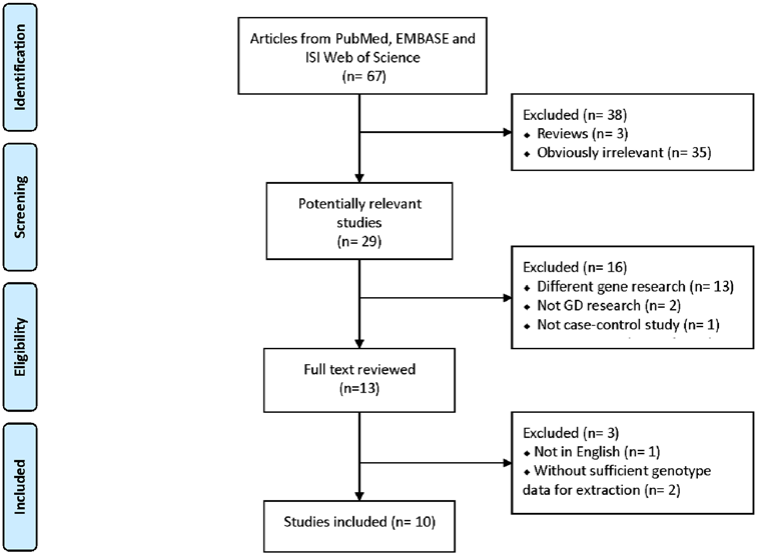
PRISMA flow diagram showing the search strategy

A total of 67 publications were characterized based on our keyword search. After screening the titles and abstracts, 35 studies were identified as irrelevant, and 3 articles were characterized as reviews. Additionally, 16 studies were excluded because 13 of the articles focussed on different genes. Another three articles were excluded because they were not on GD research (two studies) or were not case–control studies (one study). Amongst the remaining thirteen publications, three studies were also rejected as they either failed to provide detailed genotyping information (two articles) or were published in non-English journals (one study) (Figure 2).

### Characteristics of the selected datasets

A total of ten case–control datasets were identified based on our selection criteria. Of these, nine studies were conducted for the rs1800629 polymorphism which included 1980 GD patients and 2636 controls, while six studies were carried out for the rs361525 polymorphism which involved 1869 patients and 2300 controls. The principal characteristics and genotype distributions of the identified studies are shown in Table 1. For SNP rs1800629, six studies were found from Asian [11–16], and three studies were from European population [8,9,17]. For the rs361525 polymorphism, there were four studies originating from Asian [12–14,16], while the rest two studies were from European population [6,8]. Genotypic distribution for both rs1800629 and rs361525 in controls was in consistent with HWE (*P*>0.05) except for the four datasets highlighted in bold (Table 1). Each study was scored based on the NOS, as shown in Table 2. These nine case–control studies scored 7–8, indicating sufficient quality for inclusion in the meta-analysis.

**Table 1:** Summary of datasets included for meta-analysis

Study ID	Author	Year	Ethnicity	Genotyping method	Study design	Case/control	SNP loci	GD patient			Healthy control			*p*HWE
								GG	GA	AA	GG	GA	AA	
1	Duraes et al. [9]	2014	European	Taqman	CC	111/735	rs1800629	72	34	5	562	156	17	0.122
2	Kutluturk et al. [11]	2013	Asian	PCR-SSP	CC	100/124	rs1800629	73	24	3	103	15	6	**0.000**
3	Jurecka-Lubieniecka et al. [17]	2013	European	PCR-RFLP	CC	555/341	rs1800629	299	231	25	259	71	11	**0.032**
4	Anvari et al. [12]	2010	Asian	PCR-SSP	CC	105/137	rs1800629	56	44	5	98	39	0	0.052
							rs361525	74	33	0	79	57	1	**0.007**
5	Gu et al. [13]	2010	Asian	MassArray™	CC	426/315	rs1800629	368	56	2	263	51	1	0.369
							rs361525	408	20	0	281	34	0	0.311
6	Shiau et al. [14]	2007	Asian	PCR-RFLP	CC	187/101	rs1800629	168	16	3	77	24	0	0.175
							rs361525	50	70	3	186	3	0	0.912
7	Chen et al. [15]	2005	Asian	PCR-RFLP	CC	95/60	rs1800629	85	10	0	49	9	2	0.083
8	Bednarczuk et al. [8]	2004	European	PCR-SSP	CC	228/248	rs1800629	122	96	10	172	72	4	0.25
							rs361525	220	8	0	225	22	1	0.563
9	Simmonds et al. [6]	2004	European	PCR-RFLP	CC	810/836	rs361525	660	145	5	727	105	4	0.92
10	Kamizono et al. [16]	2000	Asian	PCR-SSOP	CC	173/575	rs1800629	169	4	0	556	18	1	**0.04**
							rs361525	166	7	0	552	23	0	0.62

Abbreviations: CC, case/control; PCR-SSOP, PCR-sequence specific oligonucleotide polymorphism; PCR-RFLP, PCR-restriction fragment length polymorphism; PCR-SSP, PCR-sequence specific primer.

**Table 2:** Quality assessments of case–control studies according to the NOS

Study ID	Authors	Year	Selection				Comparability		Exposure			Total score
			a	b	c	d	e	f	g	h	i	
1	Duraes et al. [9]	2014	*	*	/	*	*	/	*	*	*	7
2	Kutluturk et al. [11]	2013	*	*	/	*	*	/	*	*	*	7
3	Jurecka-Lubieniecka et al. [17]	2013	*	*	/	*	*	/	*	*	*	7
4	Anvari et al. [12]	2010	*	*	/	*	*	/	*	*	*	7
5	Gu et al. [13]	2010	*	*	/	*	*	*	*	*	*	8
6	Shiau et al. [14]	2007	*	*	/	*	*	/	*	*	*	7
7	Chen et al. [15]	2005	*	*	/	*	*	*	*	*	*	8
8	Bednarczuk et al. [8]	2004	*	*	/	/	*	/	*	*	*	6
9	Simmonds et al. [6]	2004	*	*	*	*	*	/	*	*	*	8
10	Kamizono et al. [16]	2000	*	*	/	*	*	/	*	*	*	7

**Publication quality check list**

***Selection***: **a**: Is the case definition adequate? **b:** Representativeness of the cases. **c:** Selection of controls; **d:** Definition of controls.

***Comparability:*** **e:** Study controls for ethnicity. **f:** Study controls for any additional factor.

***Exposure:*** **g:** Ascertainment of exposure. **h:** Same method of ascertainment for cases and controls. **i:** Non-response rate.

The asterisks (*) represent the stars in the NOS assessment.

### Association between *TNF-α* gene polymorphism and GD

Meta-analysis for the promoter SNP rs1800629 was carried out by including 1980 GD patients and 2636 controls. A significant association was characterized between the rs1800629 polymorphism and GD in the homozygous model (AA compared with GG: OR = 1.97, 95% CI = 1.27–3.06, *P*=0.002) and recessive model (AA compared with GA + GG: OR = 1.62, 95% CI = 1.04–2.50, *P*=0.03) (Table 3). For analysis of ethnic stratification, we divided the datasets into two subgroups, Asian and European. GD susceptibility was significantly detected in European population in all genetic models. In sharp contrast, no significant association could be detected in Asian population (Table 4). Next, we conducted a meta-analysis for another promoter SNP rs361525, in which we have included the above identified five datasets (1869 patients and 2300 controls in total). However, SNP rs361525 did not show a significant association with GD in any genetic model before and after ethnicity stratification (Tables 3 and 4). Of note, our meta-analysis for SNP rs1800629 and rs361525 was hampered by the presence of genetic heterogeneity, which could be due to the differences of ethnicities and gene–environmental interactions.

**Table 3:** Results for meta-analysis of *TNF-α* polymorphisms with GD risk

SNPs	OR (95% CI)	*P*-value	Test of heterogeneity		*p* for publication bias^1^
			*I^2^*	*P*-value	
**rs1800629 (G > A)**					
AA compared with GG	1.97 [1.27, 3.06]	**0.002**	10.9%	0.34	0.71
GA compared with GG	1.26 [0.80, 1.98]	0.33	85.2%	0.00	0.13
AA + GA compared with GG	1.25 [0.81, 1.94]	0.32	85.0%	0.00	0.08
AA compared with GA + GG	1.62 [1.04, 2.50]	**0.03**	4.4%	0.40	0.99
A compared with G allele	1.20 [0.84, 1.71]	0.31	81.9%	0.00	0.04
**rs361525 (G > A)**					
AA compared with GG	1.67 [0.67, 4.24]	0.266	42.0%	0.16	0.99
GA compared with GG	1.38 [0.51, 3.74]	0.522	94.0%	0.00	0.91
AA + GA compared with GG	1.38 [0.51, 3.76]	0.530	94.2%	0.00	0.92
AA compared with GA + GG	1.47 [0.57, 3.80]	0.427	3.7%	0.37	0.94
A compared with G allele	1.28 [0.52, 3.16]	0.587	93.5%	0.00	0.96

1 Egger’s test was performed to assess publication bias.

*P* < 0.05 was considered statistically significant.

**Table 4:** Subgroup analysis of rs1800629 and rs361525 in *TNF-α*

Polymorphism	Genetic model	Ethnicity	Number of datasets	OR (95% CI)	*P-*value	Test of heterogeneity	
						*I*^*2*^	*P*-value
**rs1800629**	AA compared with GG	Asian	6	1.40 [0.64, 3.06]	0.396	27.3%	0.230
		European	3	2.31 [1.35, 3.95]	**0.002**	0.0%	0.713
	GA compared with GG	Asian	6	0.91 [0.50, 1.69]	0.772	79.9%	0.000
		European	3	2.14 [1.55, 2.95]	**0.000**	53.8%	0.115
	AA + GA compared with GG	Asian	6	0.90 [0.50, 1.61]	0.720	79.3%	0.000
		European	3	2.18 [1.67, 2.84]	**0.000**	38.0%	0.199
	AA compared with GA + GG	Asian	6	1.32 [0.60, 2.87]	0.489	25.3%	0.245
		European	3	1.78 [1.04, 3.02]	**0.000**	0.0%	0.605
	A compared with G	Asian	6	0.89 [0.53, 1.48]	0.647	77.7%	0.000
		European	3	1.9 [1.60, 2.28]	**0.000**	0.0%	0.46
**rs361525**	AA compared with GG	Asian	4	3.61 [0.75, 17.3]	0.109	72.8%	0.055
		European	2	1.09 [0.33, 3.55]	0.891	0.0%	0.429
	GA compared with GG	Asian	4	2.02 [0.32, 12.69]	0.452	96.1%	0.000
		European	2	0.80 [0.20, 3.16]	0.746	90.0%	0.002
	AA + GA compared with GG	Asian	4	2.04 [0.32, 13.07]	0.453	96.2%	0.000
		European	2	0.78 [0.19, 3.20]	0.725	90.7%	0.001
	AA compared with GA + GG	Asian	4	2.82 [0.53, 15.1]	0.225	53.4%	0.143
		European	2	1.04 [0.32, 3.41]	0.946	0.0%	0.470
	A compared with G	Asian	4	1.85 [0.35, 9.87]	0.469	95.8%	0.000
		European	2	0.76 [0.19, 3.05]	0.694	90.8%	0.001

*P* < 0.05 was considered statistically significant.

### Publication bias

**Figure 3:**
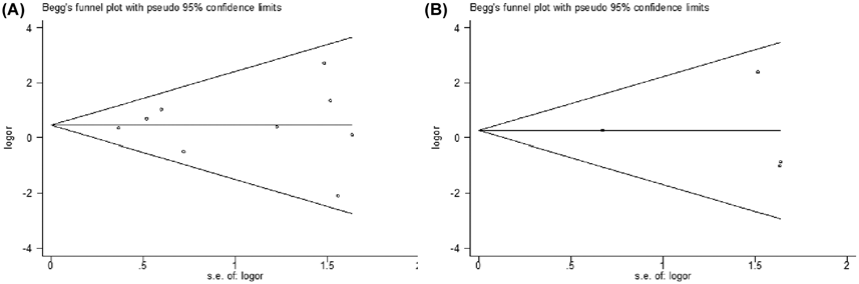
Funnel plot analysis to detect publication bias Each point represents a separate study for the indicated association. (**A**) SNP rs1800629: AA compared with (GA + GG), (**B**) SNP rs361525: AA compared with (GA + GG).

Begg’s funnel plot and Egger’s test were next conducted to assess publication bias. The shape of the funnel plots appeared to be symmetrical [SNP rs1800629: AA compared with (GA + GG); SNP rs361525: AA compared with (GA + GG)] and the Egger’s test did not show any evidence of publication bias (Figure 3). Analysis of sensitivity also revealed that results derived from our study are stable and reliable (data not shown).

## Discussion

TNF-α is an inflammatory cytokine that is produced by intrathyroidal inflammatory cells and thyroid follicular cells and plays a pivotal role in regulating immunological reactions and the development of autoimmune diseases [18]. Upon the recognition of this functional property, *TNF-α* has thus been considered to be a candidate gene for GD. Nevertheless, no consistent results have been reached so far in terms of its genetic predisposition in GD pathoetiology. To address this question, we conducted a meta-analysis with the aim of concentration on the two SNPs, G-238A (rs361525) and G-308A (rs1800629) in the promoter region. Our studies demonstrated by clear and convincing evidence that only the promoter SNP rs1800629 within the *TNF-α* gene is associated with an increased risk for developing GD. The results of our overall meta-analysis supported that only G- > A mutation at −308 in *TNF-α* was a risk factor for GD, while the other SNP did not show a significant association with GD in any genetic model. To exclude the influence of population stratification, we then divided all datasets into two subgroups, Asian population and European population. Much stronger association was noted in the European populations, while the association was undetectable in the Asian population, representing the existence of genetic heterogeneity between different ethnic groups, which could be caused by the differences of gene–environmental interactions. These results were consistent with the findings of Duraes et al. [9] in a Portuguese population and Jurecka-Lubieniecka et al. [17] in a Polish population. TNF-α is produced by monocytes, T cells, natural killer cells, and mast cells, which is an essential contributing factor for the autoimmune thyroid dysfunctions. *TNF-α* −308 A allele is associated with a higher level of *TNF-α* transcript, due to the great potency of the promoter region to activate the transcription [19,20]. Therefore, individuals carrying higher TNF-α secreting genotypes may be susceptible to GD development. *TNF-α* gene polymorphisms at position −238 is another SNP which is commonly studied. Although our meta-analysis did not detect an association between −238 and GD, it was reported that the region between −254 and −230 contains a regulatory sequence that acts as a *TNF-α* repressor site, and thus a mutation at −238 might be disrupting regulation [19,21].

Our meta-analysis has some key advantages compared with individual studies. First, to guarantee the quality of the present study, we included the most updated literature and used explicit criteria for study inclusion and a strict procedure for data extraction. Additionally, a substantial number of subjects were pooled from individual studies, which significantly increased the statistical power. However, there are several limitations in our study. First, the controls were hospital-based study in our included literatures. Compared with hospital-based study, a population-based case–control study can reduce more selection bias and have higher confidence. Second, our search was limited to published English language studies. Some potential studies which were published in other languages or unpublished have been systematically excluded. This may explain some publication bias in our meta-analysis, which may have affected the results of this meta-analysis in as far as those studies that had produced negative results might not have been published. Third, the study population is limited for meta-analysis. Considering this would lead to low statistical power, future studies with a large dataset would be necessary for fully establishing the impact on susceptibility to GD.

In summary, the results of our meta-analysis identified that only the promoter SNP rs1800629 within the *TNF-α* gene is associated with increased risk for developing GD, especially in European population. However, future studies with a large dataset focussing on addressing their functional relevance would be necessary for fully establishing their effect on GD susceptibility.

## Abbreviations

CI: confidence interval
GD: Graves’ disease
HLA: human leukocyte antigen
HWE: Hardy–Weinberg equilibrium
NOS: Newcastle–Ottawa Scale
OR: odds ratio
SNP: single-nucleotide polymorphism
TNF-α: tumor necrosis factor-α
